# Effects of Methadone on the Minimum Anesthetic Concentration of Isoflurane, and Its Effects on Heart Rate, Blood Pressure and Ventilation during Isoflurane Anesthesia in Hens (*Gallus gallus domesticus*)

**DOI:** 10.1371/journal.pone.0152546

**Published:** 2016-03-28

**Authors:** André Escobar, Rozana Wendler da Rocha, Bruno Henri Pypendop, Darcio Zangirolami Filho, Samuel Santos Sousa, Carlos Augusto Araújo Valadão

**Affiliations:** 1 Department of Veterinary Clinics and Surgery, School of Agricultural and Veterinarian Sciences, Sao Paulo State University, Jaboticabal, SP, Brazil; 2 Department of Surgical and Radiological Sciences, School of Veterinary Medicine, University of California Davis, Davis, California, United States of America; University of Bari, ITALY

## Abstract

The aim of this study was to measure the temporal effects of intramuscular methadone administration on the minimum anesthetic concentration (MAC) of isoflurane in hens, and to evaluate the effects of the isoflurane-methadone combination on heart rate and rhythm, blood pressure and ventilation. Thirteen healthy adult hens weighing 1.7 ± 0.2 kg were used. The MAC of isoflurane was determined in each individual using the bracketing method. Subsequently, the reduction in isoflurane MAC produced by methadone (3 or 6 mg kg^-1^, IM) was determined by the up-and-down method. Stimulation was applied at 15 and 30 minutes, and at 45 minutes if the bird had not moved at 30 minutes. Isoflurane MAC reduction was calculated at each time point using logistic regression. After a washout period, birds were anesthetized with isoflurane and methadone, 6 mg kg^-1^ IM was administered. Heart rate and rhythm, respiratory rate, blood gas values and invasive blood pressure were measured at 1.0 and 0.7 isoflurane MAC, and during 45 minutes after administration of methadone once birds were anesthetized with 0.7 isoflurane MAC. Fifteen minutes after administration of 3 mg kg^-1^ of methadone, isoflurane MAC was reduced by 2 (-9 to 13)% [logistic regression estimate (95% Wald confidence interval)]. Administration of 6 mg kg^-1^ of methadone decreased isoflurane MAC by 29 (11 to 46)%, 27 (-3 to 56)% and 10 (-8 to 28)% after 15, 30 and 45 minutes, respectively. Methadone (6 mg kg^-1^) induced atrioventricular block in three animals and ventricular premature contractions in two. Methadone caused an increase in arterial blood pressure and arterial partial pressure of carbon dioxide, while heart rate and pH decreased. Methadone, 6 mg kg^-1^ IM significantly reduced isoflurane MAC by 30% in hens 15 minutes after administration. At this dose, methadone caused mild respiratory acidosis and increase in systemic blood pressure.

## Introduction

The pharmacodynamics of opioids in avian patients are still understudied; most investigations on antinociceptive and immobilizing effects of opioids are with butorphanol or buprenorphine [1–5], which are not full agonist at μ-opioid receptors [6]. The butorphanol dosage that decreases the minimum anesthetic concentration (MAC) of sevoflurane is unsafe and has short-lived effects in guineafowl [7] and the analgesic effect of butorphanol in birds is controversial [2,3]. On the other hand, morphine and fentanyl, which are full agonists at μ-opioid receptors, reduced isoflurane MAC by more than 50% in chickens and red-tailed hawks, respectively [8,9].

Methadone is a μ-opioid receptor agonist [10] and also an antagonist of N-methyl-D-aspartate (NMDA) receptors [11]. Those two complementary mechanisms may potentially contribute to an analgesic and anesthetic-sparing effect in birds. In dogs, intravenous methadone administration reduced the MAC of isoflurane by 30% for at least five hours [12]. Intravenous methadone administration in cats decreased the MAC of isoflurane by 25% at approximately 30 minutes after administration, but this effect was short-lived [13]. In rats, methadone induced a dose-dependent reduction in sevoflurane MAC for at least 6 hours, and the high dose studied decreased MAC by 100% [14].

To the author’s knowledge there are no studies assessing the anesthetic-sparing effect of methadone in birds, or evaluating the cardiorespiratory effects of equipotent concentrations of isoflurane alone or combined with methadone. The purpose of this study was to determine the reduction in minimum anesthetic concentration of isoflurane in hens (*Gallus gallus domesticus*) induced by methadone administered at 2 different doses, and to characterize some cardiorespiratory effects of the dose producing an anesthetic-sparing effect. We hypothesized that methadone would reduce the MAC of isoflurane in hens in a dose-dependent manner, and that the methadone-isoflurane combination would result in less depression of heart rate and rhythm, blood pressure and ventilation than an equipotent concentration of isoflurane alone.

## Materials and Methods

This study was approved by the Committee on the Ethics of Animal Use of the Sao Paulo State University in Jaboticabal, Brazil (CEUA protocol 008078/13).

### Phase 1—Determination of the Effects of Methadone on the MAC of Isoflurane

#### Animals

Thirteen 8–12 month old ISA Brown poultry chickens weighing 1.7 ± 0.2 kg (mean±SD) were used in this study. Water and poultry feed were provided ad libitum and were not withheld from birds before the study. Birds were housed in a stall (3 X 3 X 4 m) and were considered healthy based on physical examination and a complete blood count and biochemistry profile. No artificial lighting was used, and temperature was controlled.

#### Study Preparation

General anesthesia was induced in each animal with isoflurane (Isoforine; Cristália Produtos Químicos e Farmacêuticos Ltda, Brazil) in oxygen, using a face mask connected to a Bain circuit. Oxygen flow rate was 3 L minute^-1^ and isoflurane vaporizer setting was 5% (Isoflurane Sigma Delta Vaporizer; Penlon Inc., USA). The chicken’s trachea was intubated with a 3.0 mm endotracheal tube and the cuff was not inflated. After animals were positioned in dorsal recumbency, the oxygen flow rate was reduced to 1 L minute^-1^ and the isoflurane vaporizer setting was reduced to 1.5%. Intermittent positive pressure ventilation was initiated, with a peak inspiratory pressure of 15 cm H_2_O and an inspiration-expiration ratio of 1:3. The respiratory rate was adjusted to maintain normocapnia (end-tidal partial pressure of carbon dioxide [P_E_′CO_2_] between 30 and 40 mm Hg). End-tidal gas samples (10 mL) were collected over 7 to 10 breaths, using a glass syringe, from a 3.5F catheter (Tom Cat 3.5F Fr; Ortovet, Brazil) introduced within the lumen of the endotracheal tube, with the tip of the catheter located near the distal end of the endotracheal tube. P_E_′CO_2_ and isoflurane concentration in these samples were determined using an infrared spectrometer (DX-AJAGA-1 (AGA); Dixtal, Brazil). The anesthetic gas analyzer was calibrated before and during each experiment with room air and three calibration standards containing 0.5, 1.5 and 3% isoflurane (White Martins Gases Industriais SA, Brazil) [15]. End-tidal isoflurane values were corrected using the linear regression equation generated from the calibration standards; P_E_′CO_2_ auto-calibration was performed by the infrared spectrometer before and during the study.

Ulnar venous catheterization was performed using a 24-gauge catheter (BD Angiocath; BD, Brazil) for administration of NaCl 0.9% solution at 5 mL kg^-1^ hour^-1^ via a syringe pump (Medfusion 2010i; Medex Inc., GA, USA). Pulse rate was measured with a pulse oximeter (Dixtal 2010; Dixtal, Brazil). Systolic blood pressure was measured using a Doppler ultrasound probe (Ultrasonic Doppler flow detector model 812; Parks Medical Eletronic Inc, Brazil) positioned over the median metatarsal artery, and a cuff, the width of which was approximately 40% of the thigh circumference, positioned proximally to the ultrasound probe, and connected to a sphygomanometer. Cloacal temperature was monitored with a mercury thermometer (Veterinary Thermometer; Incoterm, Brazil) and maintained between 40 and 41°C, using a heat lamp and a circulating warm water blanket (T/Pump; Gaymar, NY, USA) as needed.

#### Determination of the MAC of isoflurane

The MAC of isoflurane was determined in each bird using the bracketing method [4]. Using this experimental design, MAC is defined as the mean of 2 consecutive isoflurane concentrations not different by more than 10–20%, one allowing movement and one preventing movement in response to a noxious stimulus [16].

Each chicken was anesthetized with a predetermined end-tidal isoflurane concentration (1.0 to 1.3%) for 15 minutes and baseline heart rate, respiratory rate, P_E_′CO_2_, end-tidal isoflurane concentration, systolic blood pressure, and cloacal temperature were recorded. End-tidal gas samples were collected in triplicate and the mean value for P_E_′CO_2_ and end-tidal isoflurane concentration were calculated and reported. A noxious electrical stimulus was then applied to the medial side of the chicken’s thigh area using a pair of subcutaneous needles connected to an electrical stimulator (SD9 Square Pulse Stimulator, Astro-Med Inc, RI, USA). Each pair of needles was replaced every two stimuli and positioned at a different site of the medial thigh skin to prevent desensitization [17]. The electrical stimulus (15V, 6.5 milliseconds, 50 Hz) was applied for 1 minute or until movement was observed (movement of the head, contralateral leg, wings or tail). End-tidal isoflurane concentration was increased or decreased by 10% if movement was observed or not, respectively. Anesthesia was maintained at the new isoflurane concentration for 15 minutes, measurements obtained as described for baseline, and the electrical stimulus was applied again. This procedure was repeated until a change in response was observed; MAC was calculated as the mean of the 2 successive concentrations, one allowing and one preventing movement. MAC was determined in triplicate and the mean of the three MAC values was reported. Local barometric pressure was estimated to be 716 mm Hg, and MAC values were corrected to sea-level barometric pressure using the formula [18]:

$$\text{MAC }\left(\text{\%}\right)\text{ at sea level}=\text{Measured MAC }\left(\text{\%}\right)\;\;x\;\;\left(\frac{716}{760}\right)$$

#### Determination of the effects of methadone on the MAC of isoflurane

Immediately after determination of the individual isoflurane MAC (i.e. during the same anesthetic event), the effect of methadone, 3 mg kg^-1^ IM, on the MAC of isoflurane was determined using a quantal design [4,19]. This design allows the reduction in isoflurane MAC to be determined at predetermined time points (i.e. 15-minutes intervals). Contrary to the bracketing method, with the quantal method, each animal’s response (movement or no movement) is assessed at a single anesthetic concentration; data from the whole study sample were fitted to a logistic model to calculate the reduction in isoflurane MAC in the chicken population at specific intervals after methadone administration.

In the first bird, end-tidal isoflurane concentration was reduced to 0.7 times this individual’s MAC and maintained constant for 15 minutes. Methadone (Mytedom; Cristália Produtos Químicos e Farmacêuticos Ltda, Brazil), 3 mg kg^-1^, was injected in the pectoral muscle. Prior to each noxious stimulation, heart rate, respiratory rate, P_E_′CO_2_, end-tidal isoflurane concentration, systolic blood pressure, and cloacal temperature were recorded as described above. A noxious electrical stimulus was applied as previously described 15 and 30 minutes after the administration of methadone, and the response (movement or no movement) was recorded. Stimuli were always applied at 15 and 30 minutes due to lack of pharmacokinetic studies for methadone in chickens. If no movement was observed after 30 minutes, the electrical stimulus was applied every 15 minutes until the animal moved. Isoflurane concentration was maintained constant (i.e. at 0.7 times this individual’s MAC) for the whole duration of measurements. If movement was observed at 30 minutes after methadone administration, the study for that bird was considered completed and the bird was allowed to recover from anesthesia. If a positive response (movement) in response to noxious electrical stimulation was observed after 30 minutes of injection, a positive response was assumed at subsequent time points.

The isoflurane concentration in the subsequent birds was selected according to the response (movement or no movement) of the previous bird 15 minutes after methadone administration. If a negative response (no movement) to stimulation was observed in the previous bird at any time point after methadone administration (i.e. 15, 30, 45, etc. minutes), end-tidal isoflurane concentration for the next bird was decreased by 0.1 times the bird’s individual isoflurane MAC (e.g. 0.6 MAC for the second bird if a negative response was observed at any time point in the first bird). If a positive response (movement) to stimulation was observed in the previous bird both 15 and 30 minutes following methadone administration, the end-tidal isoflurane concentration for the next bird was increased by 0.1 times the bird’s individual isoflurane MAC (e.g. 0.8 MAC for the second bird if a positive response was observed at 15 and 30 minutes after methadone administration in the first bird). The selected isoflurane concentration was maintained constant for the whole duration of measurements. Measurements and stimulations were conducted, and responses were recorded as described for the first bird. A single isoflurane concentration was studied in each bird. Because MAC is defined as the concentration at which the probability of movement equals the probability of no movement, if the concentration selected in the quantal study for a particular individual was equal to 1.0 MAC, the electrical stimulation was performed at 15 minutes after methadone administration only, and an identical response was assumed at the subsequent time points. In case the concentration selected was higher than 1.0 MAC, the studied was terminated if the bird did not move at 30 minutes after methadone administration.

At least one week after the first anesthetic event (washout period), the determination of the effect of methadone on the MAC of isoflurane was repeated using identical methods, with the exception that the dose of methadone administered was 6 mg kg^-1^ IM.

#### Anesthetic times

Time intervals were calculated as follows: induction time (from initial administration of isoflurane to intubation), instrumentation time (from endotracheal intubation to completion of placement of monitoring instruments), time to MAC determination (from end of instrumentation to end of determination of the individual’s MAC), study time (from methadone administration to the discontinuation of isoflurane administration), time to extubation (from discontinuation of isoflurane administration to extubation), and time to stand (from discontinuation of isoflurane administration to the bird assuming a standing position).

#### Statistical Analysis

Physiologic variables, anesthetic times and isoflurane MAC values were analyzed for normality using the Shapiro-Wilk test. Mean ± SD are reported for normally distributed data. Physiologic variables obtained at time points used for MAC determination (i.e. physiologic variables at 1.0 MAC of isoflurane) were compared to data after methadone administration using one-way ANOVA, followed by the Tukey test for pairwise comparisons. Time to extubation and time to stand in the 3 and 6 mg kg^-1^ group were compared using the unpaired t-test or the Mann-Whitney test for normally and non-normally distributed data, respectively. Significance level was set at 5%.

To calculate the isoflurane MAC reduction after 3 and 6 mg kg^-1^ of methadone, calculated MAC reduction corresponding to the selected isoflurane concentration in each individual was fitted to a logistic model at each time point. The value yielding a probability of 0.5 for MAC reduction was calculated and is reported, with its corresponding 95% Wald confidence interval, and considered to be the MAC reduction produced by methadone for that time point and methadone dose. MAC reduction was considered statistically significant if the value was positive and the 95% confidence interval did not include 0.

### Phase 2—Evaluation of the Cardiorespiratory Effects of Methadone Isoflurane-Anesthetized Chickens

#### Animals

Eight of the 13 ISA Brown poultry chickens used in phase 1, weighing 1.9 ± 0.1 kg and aged 15 to 18 months were used. Housing and feeding were identical as in phase 1. The MAC of isoflurane had been determined in each individual chicken in phase 1.

#### Study preparation

Animals were preoxygenated for one minute, using a face mask connected to a Bain circuit with an oxygen flow rate of 3 L minute^-1^. Anesthesia was induced with isoflurane in oxygen, using the face mask and Bain circuit, with identical oxygen flow rate, and an initial vaporizer setting of 5%. The chickens’ trachea was intubated with a 3.0 mm endotracheal tube, which cuff was not inflated. Birds were positioned in dorsal recumbency, and the endotracheal tube was connected to the Bain circuit. The oxygen flow rate was reduced to 1 L minute^-1^, and the end-tidal isoflurane was adjusted to 1.0 times the individual’s isoflurane MAC. Animals were allowed to breathe spontaneously for the duration of the study. Sampling of end-tidal gases, administration of fluids and gas analyzer calibration were performed as described for Phase 1.

Electrocardiogram (ECG) electrodes were attached to the skin at the base of both wings and at the mid-thigh level and a lead II ECG (ECGPC; TEB, Brazil) was recorded. Cloacal temperature was maintained between 40° and 41°C as described above. A 24-gauge catheter was inserted in the ulnar artery for measurement of arterial blood pressure by means of a pressure transducer (Dixtal 2010; Dixtal, Brazil). Calibration of the transducer was verified using a mercury column. The transducer was positioned and zeroed at the level of bird’s sternal extremity of the coracoid bone. Approximately 0.5 mL of arterial blood was collected in a syringe containing sodium heparin, and the pH, partial pressure of oxygen (PaO_2_) and carbon dioxide (PaCO_2_), sodium, potassium and chloride concentrations were immediately measured (OMINI C; Roche Diagnostics, Brazil). In addition, bicarbonate concentration and base excess (BE) were calculated using standard equations. Blood gas measurements were adjusted for body temperature using a standard equation.

#### Evaluation of cardiorespiratory, acid-base and electrolyte effects

Baseline data (heart rate, heart rhythm, respiratory rate, body temperature, systolic, diastolic and mean arterial blood pressure, end-tidal partial pressure of carbon dioxide, and arterial pH, PO_2_, PCO_2_, and sodium, potassium and chloride concentrations) were collected 30 minutes after induction of anesthesia (T1MAC), when animals had been anesthetized at 1.0 times their individual isoflurane MAC for a minimum of 15 minutes. After baseline data had been obtained, end-tidal isoflurane concentration was decreased to 0.7 individual MAC and was kept at that concentration for the remainder of the study. Measurements were repeated as above after 15 minutes at the new isoflurane concentration (T0.7MAC). Methadone hydrochloride (6 mg kg^-1^) was administered in the pectoral muscle and measurements were repeated as previously at 1 (T1), 5 (T5), 10 (T10), 15 (T15), 30 (T30), and 45 (T45) minutes after administration.

Following completion of the study, instruments were removed and animals were allowed to recover from anesthesia. They received oxygen supplementation until extubation.

#### Statistical Analysis

The Shapiro-Wilk test was used to verify normal distribution of the data. Data obtained at T1MAC and T0.7MAC were compared to data obtained after methadone administration using a repeated-measures ANOVA, followed by Tukey’s test for pairwise comparisons where appropriate. Significance level of all tests was set at 5%. Data are reported as mean±SD.

## Results

### Phase 1

**Fig 1:**
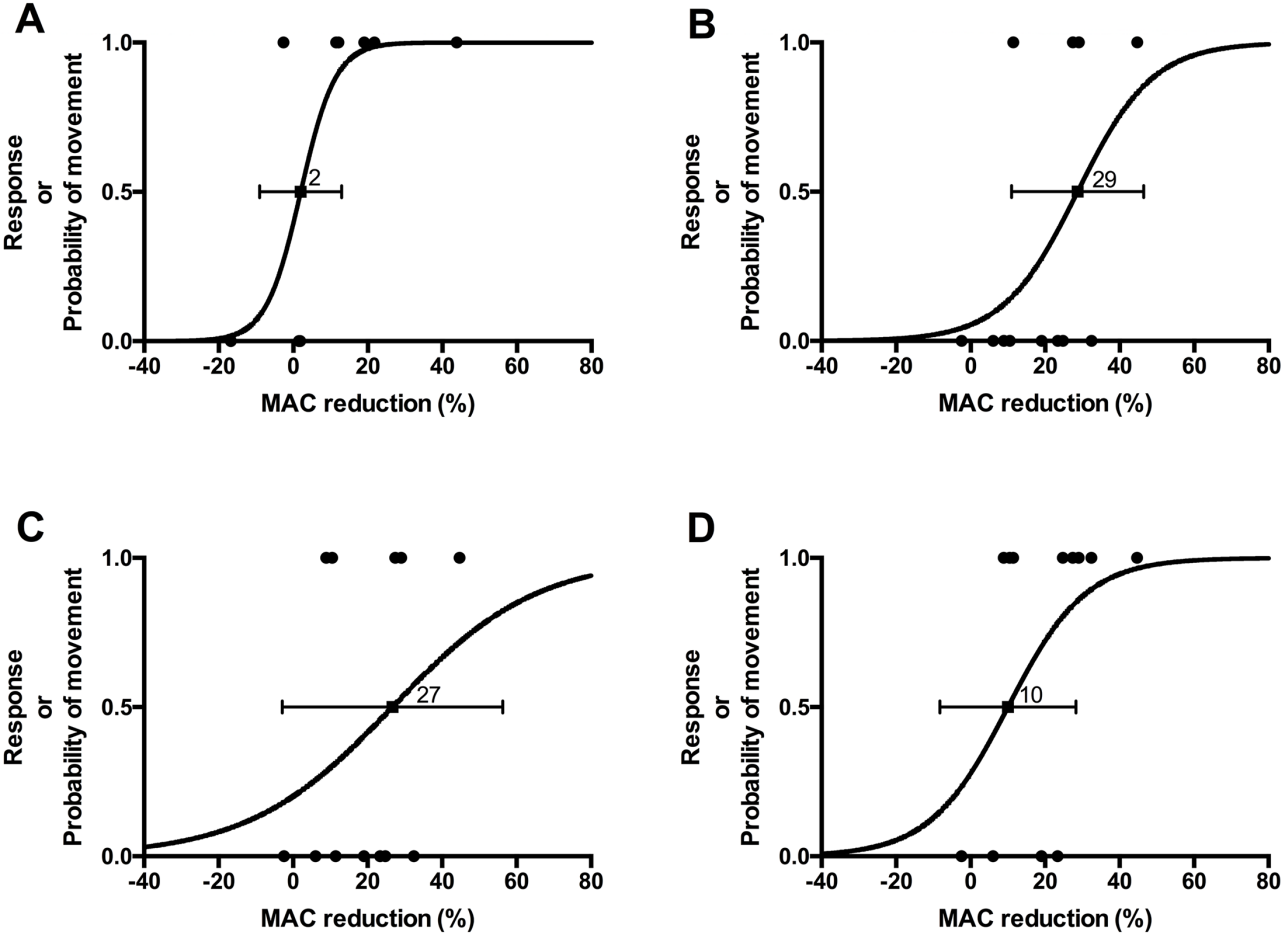
Predicted probability of MAC reduction obtained by logistic regression (line) of observed responses (closed circles) to noxious electrical stimulation in chickens anesthetized with various end-tidal concentrations of isoflurane (presented as % change from their individual isoflurane MAC), following intramuscular administration of 3 (n = 9) or 6 (n = 12) mg kg^-1^ methadone. A—Data obtained 15 minutes after IM administration of 3 mg kg^-1^ methadone. B—Data obtained 15 minutes after IM administration of 6 mg kg^-1^ methadone. C—Data obtained 30 minutes after IM administration of 6 mg kg^-1^ methadone. D—Data obtained 45 minutes after IM administration of 6 mg kg^-1^ methadone. The closed square shows the predicted MAC reduction (the label represents the value in %), and the error bar represents the 95% Wald confidence interval. The 95% confidence interval does not include 0 for the 6 mg kg^-1^ group at 15 min only, and MAC reduction was therefore considered significant only in that group at that time point. Note that some data points may be superimposed.

Using the bracketing method, mean ± SD isoflurane MAC determined for the thirteen chickens was 1.1 ± 0.1%. Nine and 12 chickens were used in the 3 and 6 mg kg^-1^ methadone groups, respectively Methadone, 3 mg kg^-1^ IM, reduced isoflurane MAC by 2 (-9 to 13)% 15 minutes after injection (Fig 1); the change was not significant. Three independent crossover events (successive independent chickens having different responses (movement or no movement) to stimulation) were observed. Data after 30 minutes of injection were identical. All chickens that were anesthetized with less than 1.0 MAC moved during electrical stimulation at 30 minutes after injection (two chickens were administered isoflurane at 1.0 MAC and the study was completed at 15 minutes after injection of methadone; one chicken was anesthetized with more than 1.0 MAC and the study terminated at 30 minutes after injection because it did not move). Following IM administration of 6 mg kg^-1^ of methadone, isoflurane MAC decreased by 29 (11 to 46)%, 27 (-3 to 56)% and 10 (-8 to 28)% 15, 30 and 45 minutes after methadone administration, respectively (Fig 1). The MAC reduction was considered statistically significant at 15 minutes after methadone administration only. Four crossover events were observed at each of these time points. Thirty minutes after injection of methadone, five chickens moved; two were anesthetized with 1.0 isoflurane MAC, and the study was therefore completed for them. Five chickens were tested 45 minutes after injection of methadone and three moved; two chickens were tested 60, 75, 90 and 105 minutes after injection of methadone and one moved at 105 minutes; the remaining chicken was tested at 120, 135 and 150 minutes after injection of methadone and moved at 150 minutes. Reduction of isoflurane MAC after 45 minutes could not be estimated due to the low number of chickens that did not move. One bird developed cloacal prolapse and rupture and was euthanatized during the study of the 6 mg kg^-1^ dose.

Measurements of cardiorespiratory variables during MAC determination and after administration of methadone are summarized in Table 1. There were no changes in any variable after methadone administration compared to baseline. Median (range) induction and instrumentation times for all anesthetic procedures were 2 (1 to 7) and 11 (6 to 24) minutes, respectively. Median time to MAC determination was 109 (87 to 179) minutes for the 13 chickens. Median study time was 21 (17 to 34) and 32 (20 to 49) minutes for the 3 and 6 mg kg^-1^ methadone groups, respectively. The difference was statistically significant (P = 0.029). Median time to extubation in the 3 and 6 mg kg^-1^ methadone groups was 5 (1 to 13) and 9 (2 to 43) minutes, respectively; the difference was not significant. One animal in the 3 mg kg^-1^ methadone group was extubated more than 10 minutes (13 minutes) after the end of anesthetic administration. Five animals in the 6 mg kg^-1^ methadone group were extubated more than 20 minutes (22, 26, 28, 34 and 43 minutes) after the end of anesthetic administration. There was no difference in median time to stand between the 3 mg kg^-1^ [51 (11 to 79) minutes] and 6 mg kg^-1^ [46 (12 to 119) minutes] methadone groups. Five animals in the 3 mg kg^-1^ methadone group took more than 50 minutes (51, 51, 55, 62 and 79 minutes) to stand after the end of anesthetic administration. Five animals in the 6 mg kg^-1^ methadone group took more than 50 minutes (61, 76, 80, 93 and 119 minutes) to stand after the end of anesthetic administration.

**Table 1:** Temperature and selected cardiorespiratory variables in hens anesthetized with isoflurane at baseline (measurement obtained at time points used for MAC determination, i.e. at isoflurane concentration close to 1.0 MAC), 15 minutes after IM administration of 3 mg kg^-1^ methadone, and 15, 30 and 45 minutes after IM administration of 6 mg kg^-1^ methadone.

Variable	Baseline at MAC, n = 13	3 mg kg^-1^, 15 min, n = 9	6 mg kg^-1^, 15 min, n = 12	6 mg kg^-1^, 30 min, n = 12	6 mg kg^-1^, 45 min, n = 5
**Heart rate (beats min^-1^)**	202 ± 44	190 ± 35	178 ± 55	183 ± 56	184 ± 38
**Indirect blood pressure (mm Hg)**	92 ± 7	95 ± 12	99 ± 12	100 ± 14	103 ± 16
**End-tidal partial pressure of carbon dioxide (P_ET_CO_2_ mm Hg)**	34 ± 3	35 ± 7	33 ± 6	35 ± 8	36 ± 5
**Temperature (°C)**	40.6 ± 0.2	40.7 ± 0.2	40.5 ± 0.2	40.4 ± 0.3	40.4 ± 0.3

Measurements were recorded immediately before application of the electrical stimulus used for MAC determinations. n = number of chickens.

### Phase 2

Mean ± SD instrumentation time was 19 ± 8 minutes. Mean ± SD monitored variables are summarized in Table 2. Atrioventricular block and ventricular premature contractions were observed following methadone administration in 3 and 2 chickens, respectively.

**Table 2:** Mean ± SD cardiorespiratory, acid-base and arterial electrolyte variables in hens anesthetized with isoflurane at 1.0 MAC (T1MAC), and at 0.7 MAC before (T0.7MAC) and after IM methadone (6 mg kg^-1^) administration (T1 to T45).

Variable	Time Point							
	T1MAC	T0.7MAC	T1	T5	T10	T15	T30	T45
**Heart rate (beats minute^-1^)**	206 ± 30	232 ± 60	210 ± 41	200 ± 38	187 ± 32†	185 ± 35†	206 ± 55	206 ± 51
**Respiratory rate (breaths minute^-1^)**	16 ± 2	18 ± 4	17 ± 3	16 ± 3	14 ± 3	14 ± 4	15 ± 4	16 ± 3
**End-tidal partial pressure of carbon dioxide (P_ET_CO_2_ mm Hg)**	39 ± 3	43 ± 5	45 ± 6	46 ± 9	48 ± 7*	50 ± 9*	49 ± 12*	51 ± 9*†
**Systolic arterial pressure (mmHg)**	96 ± 16	107 ± 15*	107 ± 15*	103 ± 14	105 ± 14*	106 ± 14*	109 ± 14*	111 ± 15*
**Diastolic arterial pressure (mmHg)**	80 ± 12	92 ± 15*	92 ± 15*	88 ± 14*	88 ± 13*	89 ± 13*	94 ± 15*	96 ± 16*
**Mean arterial pressure (mmHg)**	89 ± 13	101 ± 15*	101 ± 14*	97 ± 13*	98 ± 13*	99 ± 14*	103 ± 14*	105 ± 15*
**Cloacal temperature (°C)**	40.6 ± 0.2	40.6 ± 0.2	40.6 ± 0.3	40.7 ± 0.3	40.6 ± 0.3	40.5 ± 0.2	40.5 ± 0.2	40.5 ± 0.3
**pH**	7.45 ± 0.03	7.42 ± 0.07		7.39 ± 0.06*		7.38 ± 0.07*†	7.38 ± 0.06*	7.39 ± 0.04*
**PaO2 (mmHg)**	335 ± 36	327 ± 35		332 ± 38		316 ± 51	329 ± 52	337 ± 57
**PaCO2 (mmHg)**	31 ± 3	37 ± 8		39 ± 7*		42 ± 8*	44 ± 9*†	45 ± 9*†
**HCO_3_ (mmol L^-1^)**	21 ± 2	23 ± 2		23 ± 3*		24 ± 3*	25 ± 4*†	26 ± 4*†
**BE (mmol L^-1^)**	-2.16 ± 2.05	-0.97 ± 2.15		-0.85 ± 2.58		-0.46 ± 2.73	0.73 ± 3.8*	1.73 ± 3.67*†
**Na (mmol L^-1^)**	155 ± 4	157 ± 2		156 ± 2		156 ± 3	158 ± 2	157 ± 3
**K (mmol L^-1^)**	3.2 ± 0.2	3.3 ± 0.5		3.3 ± 0.4		3.4 ± 0.2	3.4 ± 0.3	3.7 ± 0.2*†
**Cl (mmol L^-1^)**	116 ± 3	117 ± 2		117 ± 3		116 ± 3	116 ± 2	115 ± 2

*Significantly different from values at TMAC (p < 0,05).

^†^Significantly different from values at T0.7MAC (p < 0,05).

For Cl and K values, n = 6.

## Discussion

This study reports the effects of methadone on the MAC of isoflurane in hens and some cardiorespiratory effects of the methadone/isoflurane combination. Mean isoflurane MAC reported in the present study (1.1%) was similar to that in other studies in chickens (1.24%, 1.15%) [8,20], ducks (1.3%) [21], thick-billed parrots (1.07%) [22] and cinereous vultures (1.06%) [23], but lower than in crested serpent eagles (1.46%) [24], pigeons (1.8%) [25], and red-tailed hawks (2.05%) [9]. Differences in MAC values in these studies can be attributed to species (i.e. genetic) differences, and to other influences, which include differences in methods, experimental errors and circadian rhythm [16]. In any case, the clinical relevance of the present study lies in the difference in isoflurane MAC before and after administration of methadone. Although there are differences in the methodology used for the baseline MAC for isoflurane and MAC after methadone administration, both quantal and bracketing designs are expected to result in similar estimates of MAC [16].

Intramuscular administration of methadone in chickens induced a reduction in isoflurane MAC only at the higher dose studied (6 mg kg^-1^), and only for a short time following IM administration. Electrical stimulation was performed in all animals at 15 and 30 minutes after methadone administration because we did not know the time to maximum plasma methadone concentration and therefore maximum effect, due to lack of pharmacokinetic data for methadone in chickens. We believe that time to maximum plasma methadone concentration was reached before 30 minutes after intramuscular administration, as reported for other opioids [26,27]. Although the higher dose of methadone decreased isoflurane MAC by 30%, this reduction was short lived as reported for butorphanol in guineafowl [4]. One explanation for this short duration is that methadone likely has a high clearance in chickens, such as reported for other opioids in birds [26,27].

Methadone decreases the isoflurane MAC in dogs [12,28], cats [13] and rats [14], but to the authors’ knowledge, the effects have not been studied in other species, including birds. In cats, the anesthetic sparing effect of methadone lasted for at least 76 minutes (15% of sevoflurane MAC reduction), and no MAC reduction was detected 122 minutes after administration [13]. However in dogs, intravenous methadone administration induced a dose-related decrease in isoflurane MAC for at least five hours [12]. The difference between the offset of effect of methadone in chickens and dogs or cats could be attributed to pharmacodynamic reasons such as differences in the opioid receptors binding affinity or efficacy in the spinal cord [29]. Duration of effect is also expected to be dose-dependent, because with higher doses, plasma and effect-site concentrations will take longer to decrease below the concentration producing an effect; given the lack of pharmacokinetic data in chickens, it is impossible to establish whether the dose used in this study produces plasma concentrations similar to the studies in other species. In addition, there are no studies reporting that methadone inhibits NMDA receptors in chickens, although in vitro studies have demonstrated this effect [11]. This may have influenced both magnitude and duration of effect.

There were no differences in time to extubation and to stand between chickens that received 3 or 6 mg kg^-1^ of methadone. However, five animals that received the higher dose were extubated over 20 minutes after the end of isoflurane administration. We did not compare these values to chickens anesthetized with isoflurane alone, but other studies in chickens have documented a mean extubation time of 3.8 ± 2.4 minutes [20]. Although the median time to extubation (9 minutes) for animals that received the highest methadone dose is not very long, some extubation times were considerably longer than in chickens anesthetized only with isoflurane. Similarly, time to stand in the present study was considerably longer than previously documented for chickens anesthetized with isoflurane alone (1.8 ± 1.5 minutes), even when the difference in calculation of the time (from extubation in the study with isoflurane alone, and from discontinuation of isoflurane administration in the present study) is taken into account [20]. At these doses, methadone seems to prolong anesthesia recovery in birds and should be used with caution in unhealthy patients.

The second part of the study compared the cardiorespiratory effects of isoflurane alone to isoflurane combined with methadone. Since methadone reduced isoflurane MAC by 30% at 15 and 30 minutes after administration, equipotency was achieved for measurements at T1MAC, T15 and T30, although the variability in MAC reduction at 30 minutes was larger. No adverse cardiorespiratory effects were detected in the first part of the study, however ventilation was controlled during this phase of the study, body temperature was maintained constant and invasive blood pressure and electrocardiogram were not monitored. In the second phase of the study, a significant decrease in heart rate, increase in blood pressure, mild hypercapnia and arrhythmias were detected. Heart rate after methadone administration was lower only when compared to T0.7MAC, but mean heart rate at this time point was higher because chickens were at a light plane of anesthesia. Blood pressure was significantly higher 15 and 30 minutes after methadone administration, compared to animals anesthetized with isoflurane only (T1MAC). Opioids usually decrease the heart rate due to an increase in vagal tone [6], and the increase in blood pressure could be associated to methadone-induced vasopressin release [30]. Alternatively, the increase in blood pressure may be related to the decrease in isoflurane concentration. Although some animals developed arrhythmias after methadone administration, no clinically relevant changes in blood pressure were detected.

Arterial potassium and base excess increased after methadone administration, however their value are still within the reference range for the species [31], and were similar to values reported for other anesthetized bird species [7,32]. There was a significant increase in PaCO_2_ and a decrease in arterial pH five minutes after administration of methadone. Respiratory acidosis has been described during isoflurane anesthesia under spontaneous ventilation in different avian species [21,24,25,32]. In addition, opioids induce respiratory depression in mammals [6], and methadone probably contributed to the respiratory acidosis reported in the present study. Although chickens experienced respiratory depression in the present study, this effect was considered mild.

This study should be interpreted in view of some limitations. First, we did not measure the plasma methadone concentration and we could not examine its relationship with the effect on isoflurane MAC. Second, the order of dose was not randomized, nor were the observers blinded to treatments. We first determined isoflurane MAC reduction for the 3 mg kg^-1^ dose in order to decide if the next dose should be higher or lower. Third, only 9 chickens were used for the MAC reduction study with 3 mg kg^-1^ methadone, and only 3 crossover events were observed. While, given the small effect observed, and the large interindividual variability, it is unlikely that studying the 12 chickens and/or obtaining 1–2 additional crossovers would largely change the results, the MAC reduction reported in this group should be interpreted with caution, as a minimum of 4 crossovers has been recommended for up-and-down studies [19]. In addition, in this group, the changes in isoflurane concentration from some individuals to the next were larger than the 0.1 MAC planned, which increases the uncertainty of the MAC estimate. Finally, the observed effect should not be interpreted as proof that methadone produces analgesia in chickens, or that a potential analgesic effect would have the same onset or offset as the immobilizing effect [33]. While immobilizing effects induced by drugs are mediated primary in the spinal cord, the analgesic effect of opioids is also mediated from action in higher centers [34].

## Conclusions

Methadone, at the dose of 6 mg kg^-1^ IM, decreased the isoflurane MAC by 30% 15 minutes after administration in hens. At this dose, in spontaneous ventilated hens, methadone produced mild respiratory acidosis, decreased heart rate and increased systemic blood pressure, and occasional cardiac arrhythmias were observed.
